# Cytokine Profiles and Antibody Response Associated to Choclo Orthohantavirus Infection

**DOI:** 10.3389/fimmu.2021.603228

**Published:** 2021-03-19

**Authors:** Tybbysay P. Salinas, Jose L. Garrido, Jacqueline R. Salazar, Publio Gonzalez, Nicole Zambrano, Francisco Fuentes-Villalobos, Felipe Bravo, Victor Fica-Leon, Alexis Salas-Burgos, Mario Calvo, Raymond Alvarez, Blas Armien, Maria Ines Barria

**Affiliations:** ^1^ Department of Microbiology, Biotechnology Center, Faculty of Biological Science, Universidad de Concepción, Concepción, Chile; ^2^ Department of Research in Emerging and Zoonotic Diseases, Gorgas Memorial Institute of Health Studies, Panama City, Panama; ^3^ Ichor Biologics LLC, New York, NY, United States; ^4^ Department of Pharmacology, Faculty of Biological Sciences, Universidad de Concepción, Concepción, Chile; ^5^ Institute of Medicine, Universidad Austral de Chile, Valdivia, Chile; ^6^ Sistema Nacional de Investigación (SIN), SENACYT, Panama City, Panama

**Keywords:** CHOV, HCPS, IL-4, IL-8, IL-10, IgG, neutralizing antibodies

## Abstract

**Background:**

New World Hantaviruses (NWHs) are the etiological agent underlying hantavirus cardiopulmonary syndrome (HCPS), a severe respiratory disease with high mortality rates in humans. In Panama, infections with *Choclo Orthohantavirus* (CHOV) cause a much milder illness characterized by higher seroprevalence and lower mortality rates. To date, the cytokine profiles and antibody responses associated with this milder form of HCPS have not been defined. Therefore, in this study, we examined immune serological profiles associated with CHOV infections.

**Methods:**

For this retrospective study, sera from fifteen individuals with acute CHOV-induced HCPS, were analyzed alongside sera from fifteen convalescent phase individuals and thirty-three asymptomatic, CHOV-seropositive individuals. Cytokine profiles were analyzed by multiplex immunoassay. Antibody subclasses, binding, and neutralization against CHOV-glycoprotein (CHOV-GP) were evaluated by ELISA, and flow cytometry.

**Results:**

High titers of IFNγ, IL-4, IL-8, and IL-10 serum cytokines were found in the acute individuals. Elevated IL-4 serum levels were found in convalescent and asymptomatic seropositive individuals. High titers of IgG1 subclass were observed across the three cohorts analyzed. Neutralizing antibody response against CHOV-GP was detectable in few acute individuals but was strong in both convalescent and asymptomatic seropositive individuals.

**Conclusion:**

A Th1/Th2 cytokine signature is characteristic during acute mild HCPS caused by CHOV infection. High expression of Th2 and IL-8 cytokines are correlated with clinical parameters in acute mild HCPS. In addition, a strong IL-4 signature is associated with different cohorts, including asymptomatic individuals. Furthermore, asymptomatic individuals presented high titers of neutralizing antibodies.

## Introduction

Hantavirus cardiopulmonary syndrome (HCPS) is a severe respiratory disease caused by infection with New World Hantaviruses (NWHs) (1). NWHs are negative-stranded RNA virus that are members of the family *Hantaviridae* within the order *Bunyavirales* (2–4). These viruses circulate worldwide in different natural hosts depending on the geographical region. The main route of transmission occurs when humans come into close contact with rodent excreta (saliva, urine, or feces) and inhale virus-contaminated aerosols (5).

In Panama, *Choclo Orthohantavirus* (CHOV) is the only pathogenic hantavirus that causes HCPS. CHOV is transmitted by the rodent *Oligoryzomys fulvescens* (6), and has an associated lethality of 16.5% (7). This is in contrast to other NWHs that circulate around the Americas, with mortality rates between 35-40%. The highest incidence of CHOV infections have been detected in the provinces of Los Santos (61.4%), Veraguas (16.2%), Cocle (13.5%), and Herrera (6.6%), which are all considered endemic regions in Panama (8). These areas also showed a high percentage of asymptomatic CHOV-seropositive individuals, which ranged from 16 to 62% depending on the geographical area (8–11).

Typically, NWH-induced HCPS is a severe disease that progresses through three phases: prodromal, cardiopulmonary, and convalescence (1, 10), with the cardiopulmonary phase characterized by severe symptoms, such as pulmonary edema, hypoxia, hypotension, and cardiogenic shock (5). However, CHOV infections cause a wide range of clinical manifestations from asymptomatic infection to more serious expressions of HCPS (9). Despite the potential range of disease severity, CHOV infections are often mild and characterized by fever, weakness, headache, myalgia, and sometimes gastrointestinal disorder (8–11). In fact, the finding of a high prevalence of seropositive population implies that a large number of CHOV infections are asymptomatic with little to no clinical symptoms and no clinical record (8, 10).

While the mechanisms behind the pathogenesis of HCPS are poorly understood, the pathogenesis is likely governed by a complex set of variables, which include factors like the efficiency of immune responses, platelet dysfunction, and deregulation of endothelial cell barrier functions (3). Furthermore, some studies suggest that the immune responses mounted against the virus it’s self may contribute to the severity of immunopathogenesis (4). Several studies have reported that NWH infection potently induces multiple proinflammatory cytokines and chemokines, with increasing levels of interferon-γ (IFNγ), tumor necrosis factor-α (TNF-α), transforming growth factor β (TGF-β), interleukin (IL) -6, IL-21, and IL-10 all correlated with increasing disease severity and pathogenesis (12–15). In particular, higher levels of IL-6, IL-10, and IFNγ have been associated with fatal outcomes in Andes virus (ANDV) infection (14, 15). In Europe and Asia, infection with Old World Hantaviruses (OWHs) cause a milder syndrome in humans known as, hemorrhagic fever with renal syndrome (HFRS). HFRS not only has a milder clinical presentation in the acute phases of disease, but also has lower mortality rates as compared to HCPS, 5-15% vs 35-40%, respectively (3). In comparing samples form HCPS and HFRS patients, one study found that HCPS patients displayed a more proinflammatory cytokine profile as compared to individuals suffering from acute HFRS (16). Altogether these finding suggest that proinflammatory cytokines may be important modulators of HCPS severity.

In addition to cytokines, humoral responses likely play a critical role in controlling HCPS *in-vivo*. It is known that the humoral immune response to hantaviruses is long-lasting (17). After infection, IgM antibody responses against the virus are rapidly elicited, while IgG titers increase later and may remain detectable for years (18, 19). Importantly, viral glycoproteins are principally involved in the induction of neutralizing antibodies, which persist at high levels for years in convalescent individuals (20).

The serum immune signature in CHOV disease has not been examined. Therefore, in order to investigate parameters in different groups of infection, a retrospective study was conducted to characterize the humoral and cytokine responses from individuals with acute CHOV infections, as well as asymptomatic and convalescent individuals from Panama. Our results showed that patients infected with CHOV exhibit a strong immune response based on the serum cytokine profile of individuals associated with different phases of the disease. Additionally, a variable antibody response against CHOV-glycoprotein (CHOV-GP) was detectable in the three cohorts under investigation.

## Materials and Methods

### Study Design and Patients

In order to obtain profiles of the immune response in individuals with CHOV disease, four cohorts were studied:

The first cohort included 15 patients with acute mild disease (samples collected between 4 and 8 days after the onset of symptoms) at the Rural Hospital of Tonosi. Human serum samples were collected between 2014–2017 and diagnosis was confirmed in the Gorgas Memorial Institute of Health Studies in Panama. Demographic characteristics and laboratory testing of the acute cohort are shown in **Table 1**. The second cohort included 15 convalescent subjects (blood collection between 2 and 6 years after disease). The third cohort included 33 asymptomatic subjects (seropositive) who presented IgG antibodies against the nucleoprotein (N) protein of hantavirus but without a history of acute respiratory disease (8). A fourth cohort composed of 25 healthy subjects (seronegative) with no detectable levels of IgG antibodies against the N protein of hantavirus was defined as the control group. Convalescent, asymptomatic, and healthy individuals’ serum samples were collected by serosurvey in 2018 for the Department of Research in Emerging and Zoonotic Diseases at the Gorgas Memorial Institute of Health Studies.

**Table 1:** Demographic characteristics and laboratory testing of individuals of the acute cohort included in the study.

Clinical Characteristics	Acute HCPS	Reference range
**n**	15	NA
**Sex, No. female/male**	4/11	NA
**Age, y, mean ± SD (n)**	39 ± 23 (15)	NA
**Laboratory data, mean ± SD (n)**		
** Hemoglobin, g/dL**	14.2 ± 1.46 (13)	12.0 – 16.0
** Hematocrit, %**	41.8 ± 3.89 (13)	35.0 – 47.0
** Leukocytes x10^9^/L**	6.34 ± 2.41 (13)	4.5 – 10.0
** Neutrophils, %**	72.4 ± 6.45 (13)	35 – 65
** Lymphocytes, %**	17.8 ± 4.26 (11)	25 – 40
** Platelets x10^9^/L**	132.9 ± 32.3 (13)	150.0 – 400.0
** Creatinine, mg/dL**	0.85 ± 0.07 (2)	0.3 – 1.1

HCPS, hantavirus cardiopulmonary syndrome; n, number of subjects with available data; NA, not applicable; SD, standard deviation.

Clinical diagnosis of acute HCPS was screened by enzyme-linked immunosorbent assay (ELISA) for the detection of hantavirus IgM and confirmed by strip immunoblot assay (SIA) and by reverse-transcription polymerase chain reaction detecting CHOV RNA (11). The qualitative detection of IgG antibodies to hantavirus in convalescence, asymptomatic, and healthy individuals’ serum was also screened by SIA (8). Serum samples were used for antibody detection against CHOV-GP and cytokine evaluation.

### Ethical Approval

Informed written consent was obtained from all adult participants and from parents or legal guardians of minors. Consent and assent forms were approved by Institutional Bioethics Committee at the Gorgas Memorial Institute of Health Studies in Panama City. Eligible participants were all adults and children above 2 years of age who permanently resided in endemic areas of Panama.

### Multiplex Assay

Cytokine levels in serum of acute HCPS patients (n= 15), asymptomatic (seropositive) individuals (n = 20), healthy donors (n= 21), and convalescent (n= 7) were measured using the Human High Sensitivity T Cell Magnetic Bead Panel from the Milliplex^®^ Map Kit (EMD Millipore). Levels of 13 cytokines were measured: granulocyte macrophage-colony stimulating factor (GM-CSF), interferon-gamma (IFNγ), interleukin (IL)-1β, IL-2, IL-4, IL-5, IL-6, IL-8, IL-10, IL-12 (p70), IL-13, IL-17A, and IL-21. All samples were assayed according to the manufacturer’s instructions.

Briefly, 50 μl of background, standards, quality controls, and two-fold sample dilution (in triplicate) were loaded into 96-well plates, after which antibody-coated beads were added. Plates were incubated overnight at 4°C on a plate shaker. After overnight incubation, well content was removed and washed twice using a handheld magnet. Then, 50 μl of biotinylated detection antibodies were added into each well and incubated for 1 hour at room temperature while shaking. Finally, 50 μl of streptavidin-phycoerythrin was added to each well and incubated for 30 min at room temperature. Plates were washed twice before resuspending the beads in sheath fluid, followed by analysis on a Luminex’s MAGPIX^®^, and the data were collected using the Luminex xPONENT^®^ software (v. 3.1). The median fluorescence intensities (MFI) were determined using a minimum of 50 beads per analyte and serum concentrations were calculated using standard curves for each analyte generated with standards provided with the kit. Heat map analysis to represent cytokine data was performed using python 3.7.4.

### ELISA

To detect serum antibodies against CHOV-GP, an ELISA using pseudoviral particles as previously described was performed (21, 22). Briefly, costar plates (Corning) were coated with viral particles pseudotyped with CHOV-GP (CHOV-pv). Pseudovirus was concentrated by a 20% sucrose cushion and ultracentrifuged at 145,000 x g and normalized using an HIV p24 ELISA (Sino Biological). 10pg/ml of pseudovirus was used to coat plates, after washing with PBS and blocking with PBS + 3% bovine serum albumin (BSA), serum samples (1/500 dilution) of 15 acute patients, 15 convalescents, 15 asymptomatic, and 10 healthy donors were added by 1 hour at 37°C. Following washes, plates were incubated with peroxidase-labeled goat anti-human IgG, IgM and IgA (Jackson ImmunoResearch). Finally, plates were developed using 3,3’5,5’ tetramethylbenzidine (TMB) (eBioscience), and sulfuric acid 2N was used for stopping the reaction. Optical density (OD) was measured at 450 nm.

### Cell Culture and Transfection

Human embryonic kidney 293T cells (293T) were cultured in Dulbecco’s modified Eagle’s medium (DMEM) (Hyclone) supplemented with 10% cosmic calf serum (CCS), 2mM L-glutamine, 100 U/ml penicillin and 100 μg/ml streptomycin (HyClone) (DMEM complete media), under standard culture conditions at 37°C with 5% CO_2_.

293T cells were transfected with a CHOV-GP expression plasmid (a commercially synthesized mammalian cell codon-optimized nucleotide sequence coding the CHOV glycoprotein, GenBank DQ285047.1) using calcium phosphate transfection method to express CHOV-GP on their surface (CHOV GPs-293T cells). After 16 hours transfection, the media was replaced with fresh DMEM complete media and incubated for 48 hours at 37°C and 5% CO_2_.

### Cell Based Anti–CHOV-GP Human Antibody Binding Assay

Serum specific IgG antibodies against CHOV-GP were detected by a cell-based assay and flow cytometry. Briefly, CHOV GPs-293T were used to detect binding of purified IgGs (10 µg/ml for IgG determination) from subjects (15 acute and 15 convalescent, 33 seropositive and 14 healthy donors). Polyclonal IgG was isolated from serum using NAb™ Protein A/G Spin Kit (Thermo Scientific) followed by desalting using Zeba spin columns (Thermo Scientific) according to manufacturer´s instructions.

Reactive cells were detected with Alexa Fluor 488 anti-human IgG antibody (Jackson Immuno Research). For IgG subclass determination, CHOV GPs-293T cells were incubated with sera (1/1000), washed, stained with anti-human -IgG1, -IgG2, -IgG3, or -IgG4 mouse antibody (Southern Biotech) and labeled with anti-mouse Alexa Fluor 488 antibody (Jackson ImmunoResearch). All antibody stainings were incubated for 1 hour at 4°C. The levels of CHOV GP-specific antibody binding were analyzed by flow cytometry (BD LSRFortessa X-20).

### Neutralization Assay

The neutralization activity was measured as previously (21). Briefly, an aliquot for each serum sample was heat-inactivated at 56°C for 30 min and polyclonal antibodies were isolated from serum as described in the previous section. Purified antibodies from 11 convalescents, 12 asymptomatic, 5 acute, and 5 healthy donors were analyzed for the presence of neutralizing antibodies by using a CHOV glycoprotein pseudovirion system (CHOV-pv). Neutralization was measured as a reduction of GFP expression in HEK293-Iβ3 cells. CHOV-pv (6.68 ng/ml) were preincubated with purified IgG (50, 10, 2, 0.4, 0.08 and 0.016 µg/ml) in triplicate for 1 hour at 37°C in 96-well culture plates and used to infect target cells. Neutralization percentage was normalized against cells incubated with CHOV-pv without IgG (control). After 16 hours of incubation, the culture medium was replaced with DMEM complete media. After 72 additional hours, cells were harvested and resuspended in 0.5% paraformaldehyde in PBS. Cells were analyzed for GFP expression by flow cytometry (BD LSRFortessa X-20). Data were analyzed using curve fitting and nonlinear regression to determine the concentration of antibodies required to inhibit 50% of viral infection (EC50) using GraphPad Prism.

### Clustering Based on Correlation of Cytokines Profiles

A novel data-driven approach was used for the analysis of cytokine profiles based on identifying cytokine modules using CytoMod (23). Briefly, cytokine concentrations (pg/ml) and laboratory data were log-transformed for analysis. Pearson’s correlation was used to assess cytokine covariation, and Tibshirani gap statistic was used to automatically determine the optimal number of modules (K) from scanning of K=13. Then, cytokine modules were constructed with optimal K=3 using hierarchical clustering on a correlation-based similarity metric; moreover, bootstrap clustering method was employed to improve group reliability. Specifically, a clustering reliability score was computed over 1,000 permutations. The score for each pair of data represents the fraction of times they clustered together across 1,000 random samplings (denominated ratio of co-clustering). The method detail is described in Cohen et al. (23).

To perform robust pairwise correlation, the clinical data were analyzed using raw values, while the cytokine level was transformed to log values. The data is not parametric and the pairwise correlation was performed with a percentage bend correlation (24) and using the Holm-Bonferroni into p-adjust values (25). The data frame manipulation was performed in pandas (26), and the statistical method applied with Pingouin python library (27).

### Statistics

GraphPad Prism software (V7.0) was used to compare and analyze data. The Mann–Whitney test was used for the comparison of 2 groups. The Kruskal-Wallis test was used for among-group comparisons. Data are expressed as mean ± standard deviation (SD), and p <0.05 were considered to be statistically significant.

## Results

### Characteristics of Individuals Infected With CHOV

**Figure 1:**
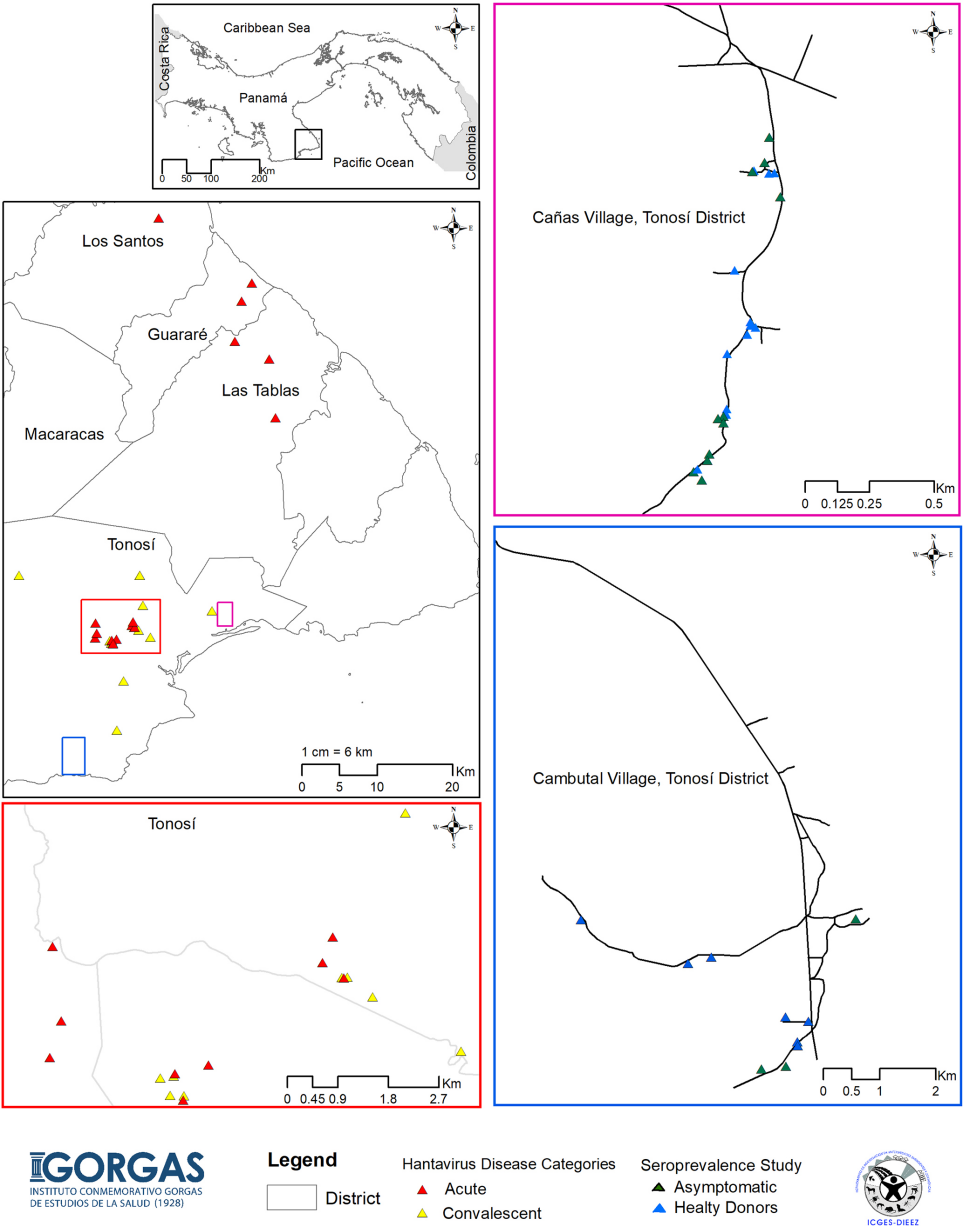
Map of the study showing HCPS endemic areas of Panama. Map of the Azuero Peninsula in Panama, HCPS endemic region, showing the location of the acute patients with HCPS (red triangle), convalescent patients (yellow triangle), asymptomatic individual (green triangle) and healthy donors (blue triangle). Large pink, blue and red squares show an enlargement of the map with more details.

In order to define the immune responses associated with CHOV infections, a total of 88 individuals were recruited from the province of Los Santos, located in the Azuero Peninsula, which is an area endemic of HCPS in Panama. Specifically, samples were collected from the districts of Tonosi (82), Guarare (2), Los Santos (1) and Las Tablas (3) (**Figure 1**). Individuals were divided into 4 different cohorts:

i) Acute phase patients consisted of 4 women and 11 men, with a mean age of 39 (SD ± 23) years.
ii) Convalescent phase individuals consisted of 10 women and 5 men, with a mean age of 46 (SD ± 20) years.
iii) Asymptomatic individuals (seropositive) included 20 women and 13 men, with a mean age of 52 (SD ± 20) years.
iv) Healthy donors (control group) consisted of 12 women and 13 men, with a mean age of 39 (SD ± 22) years.

Laboratory testing and blood parameters were determined in the acute cohort, which included hemoglobin, hematocrit, leukocyte count, percentage of neutrophils, lymphocytes, platelet count, and creatinine (**Table 1**). Most of the laboratory findings were within normal ranges. However, lymphocytes and platelet levels were decreased with respect to reference ranges. Also, an elevation in neutrophils percentage in acute patients with respect to reference ranges was detected (**Table 1**).

Overall, symptoms experienced by the acute HCPS cohort, such as weakness, myalgia, headache, and abdominal pain, were common among the group (**Table 2**).

**Table 2:** Symptoms of patients with acute hantavirus disease in Panama.

Symptoms, % (n)	Acute HCPS
*Fever >38.5 °C	100.0 (15)
**Weakness**	76.9 (13)
**Headache**	66.7 (15)
**Myalgia**	66.7 (15)
**Cough**	42.9 (14)
**Nausea**	30.8 (13)
**Vomiting**	7.7 (13)
**Diarrhea**	25.0 (12)
**Abdominal pain**	90.9 (11)

*Mean (SD) = 38.9°C ± 0.52.

HCPS, hantavirus cardiopulmonary syndrome; n, number of subjects with available data.

### Cytokines Profiles in CHOV Infection

Cytokines play key roles in both innate and adaptive immune responses. To determine the immune signatures associated with acute CHOV infections, we selected a panel of pro- and anti-inflammatory cytokines that were associated with Th1, Th2, or Th17 polarization, as well as regulatory states: Th1 immune response (IFNγ), Th2 (IL-4, IL-5, IL-13), Th17 (IL-17a) and regulatory (IL-10) immune functions. In addition, IL-21 was selected because of its ability to induce B cell differentiation and antibody production. We also selected cytokines that define neutrophils activity in lung tissue (IL-8) and markers of myeloid and dendritic cell activation regulated by innate immunity such as GM-CSF, as well as drivers of adaptive immunity IL-2, IL-6, IL-12 and IL-1β (28–30).

**Figure 2:**
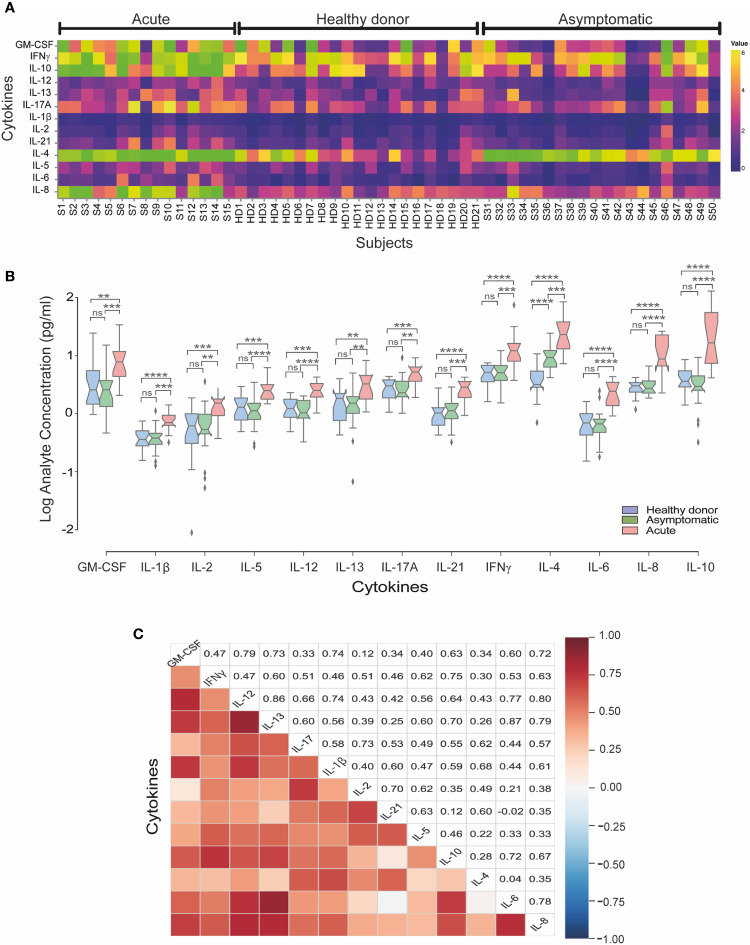
Serum cytokine levels in acute and asymptomatic cohorts in CHOV infection. **(A)** Heat map of serum cytokine concentrations for each acute patient (n=15), healthy donors (n=21) and asymptomatic individuals (n=20). Colors represent high (yellow) or low (blue) concentration. **(B)** The concentration of cytokines (log [pg/ml]) represented by a box plot diagram displayed by quartiles for each cohort: acute (red), asymptomatic (green) and healthy donors (blue). The middle line of the box plot represents the mean concentration obtained from each individual serum in triplicate. Error bars represent the SD of the mean. The mean comparison was made with Mann-Whitney U test [p scales of <0.0001 (****), <0.001 (***), <0.01 (**), ns, not significant]. **(C)** Pairwise Pearson’s correlations among log-concentrations of cytokines levels from acute samples.

To compare differences in cytokine responses across different groups, overall cytokine data was summarized and clustered in a heat map representation. We observed a specific cluster of higher levels of GM-CSF, IFNγ, IL-10, IL-4 and IL-8 cytokines in acute patients as compared to asymptomatic and healthy donor sera (**Figure 2A**). When we evaluated the mean concentrations (pg/ml) of cytokines, we observed that acutely infected individuals displayed significantly higher level of IL-8, GM-CSF, IFNγ, IL-10, IL-4 and IL-13, as compared to both the asymptomatic and healthy controls (**Figure 2B**).

Of note, IL-4 levels were significantly elevated in asymptomatic individuals as compared to healthy controls, showing that IL-4 is elevated in both acute and asymptomatic groups. In the convalescent serum samples analyzed, non-difference in most cytokines were found with only increased levels of IL-21 and IL-4 compared to healthy donors (**Supplementary Figure 1**).

To better understand the implication of cytokines profiles in the immune response in CHOV infection, a correlation analysis between cytokines was performed in the acute cohort (**Figure 2C**). Strong signature associated with IL-6, IL-8 and IL-10 was present in the acute cohort. Also, IFNγ, IL-2 and IL-10 were associated between them. Interestingly IL-4 was associated with IL-21 and IL-13 correlated with IL-6. In addition, IFNγ correlates positively with IL-12 and IL-21 even when the serological concentration of both cytokines was low.

**Figure 3:**
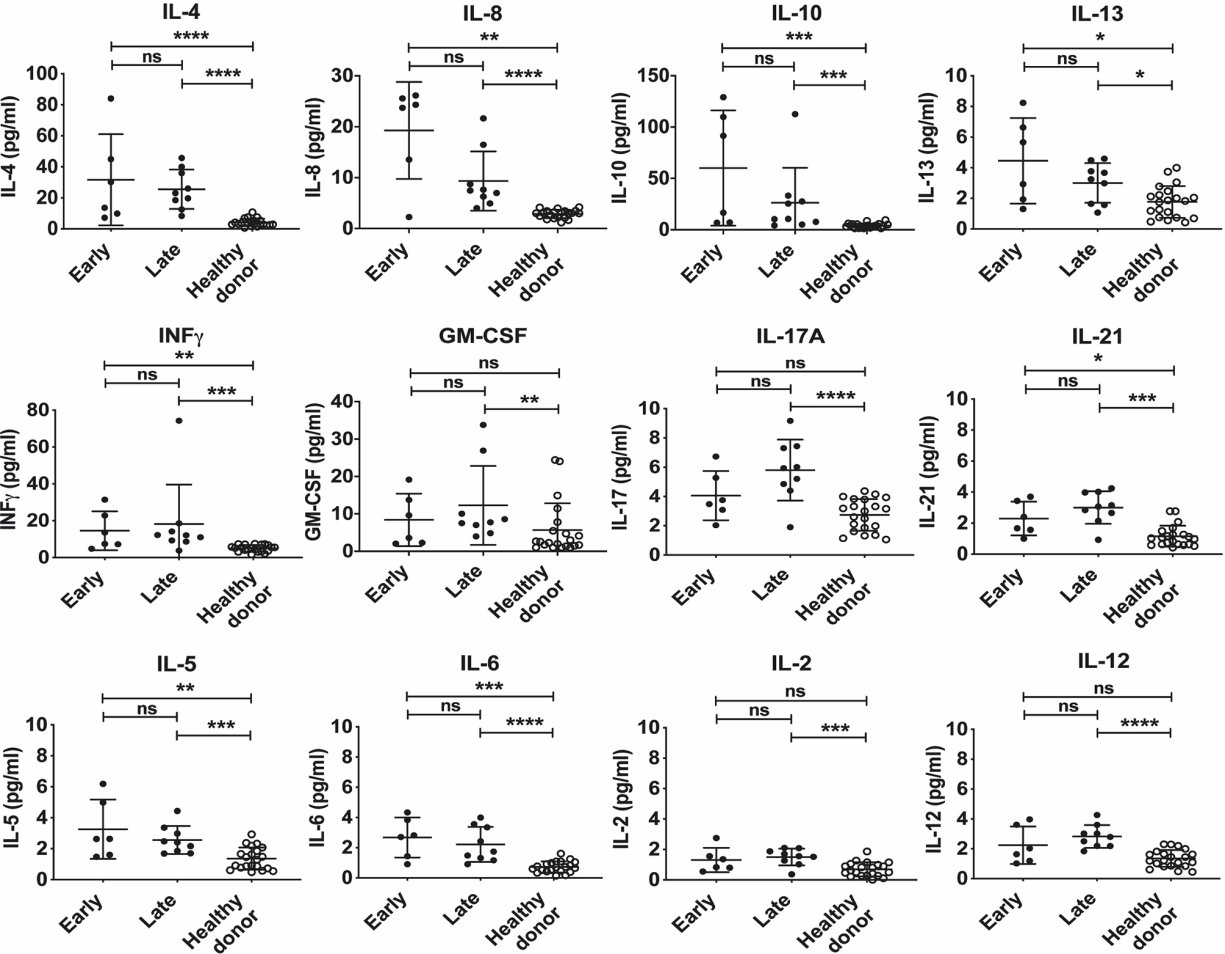
Cytokine expression levels of early and late acute patients infected with CHOV. Serum level of cytokine in acute patients (pg/ml) according to the number of days elapsed from the presentation of symptoms to sampling at “Early” presentation (<5 days, n=6) and “Late” presentation (≥ 5 days, n=9), healthy donors are presented as the control group (n=20). Each point on the graph represents an individual, and the line the mean concentration obtained from each individual in triplicate. Error bars represent the SD of the mean. The mean comparison was made with the Mann-Whitney U test [p scales of <0.0001 (****), <0.001 (***), <0.01 (**), <0.05 (*)]. ns, not significant.

In order to begin to define the cytokines that are elevated at the early and late phases of acute disease; acute samples were divided into two groups according to the number of days elapsed from symptoms presentation to sampling [early acute phase (n=6; < 5days) or late acute phase (n=9; ≥5 days)]. We observed that the mean cytokine levels (pg/ml) of IL-4 (early = 31.67 ± 29.40 vs late = 25.54 ± 12.63), IL-8 (early = 19.25 ± 9.53 vs late = 9.35 ± 5.82), IL-10 (early = 60.12 ± 56.14 vs late = 26.26 ± 34.07) and IL-13 (early = 4.45 ± 2.80 vs late = 3.01 ± 1.30) were higher in the early acute phase as compared with late acute phase, although it did not reach statistical significance. Conversely, we also noted that the mean cytokine levels of IFNγ, (early = 14.54 ± 10.54 vs late = 18.22 ± 21.40), GM-CSF (early = 8.41 ± 7.02 vs late = 12.27 ± 10.55), IL-17A (early = 4.06 ± 1.68 vs late = 5.80 ± 2.08) and IL-21 (early = 2.30 ± 1.09 vs late = 3.01 ± 1.05) were all higher in the late acute phase, as compared to the early phase, although it did not reach statistical significance. Despite finding that the differences between early and late cytokine concentrations did not reach statistical significance, we observed a trend in the profiles in both groups (**Figure 3**) suggesting a suppressive early trend immune response characterized by a Th2 profile. Finally, the levels of IL-5, IL-6, IL-2 and IL-12 were slightly increased in the late acute phase versus healthy subjects (**Figure 3**).

### Antibody Titers Against the CHOV Glycoprotein

**Figure 4:**
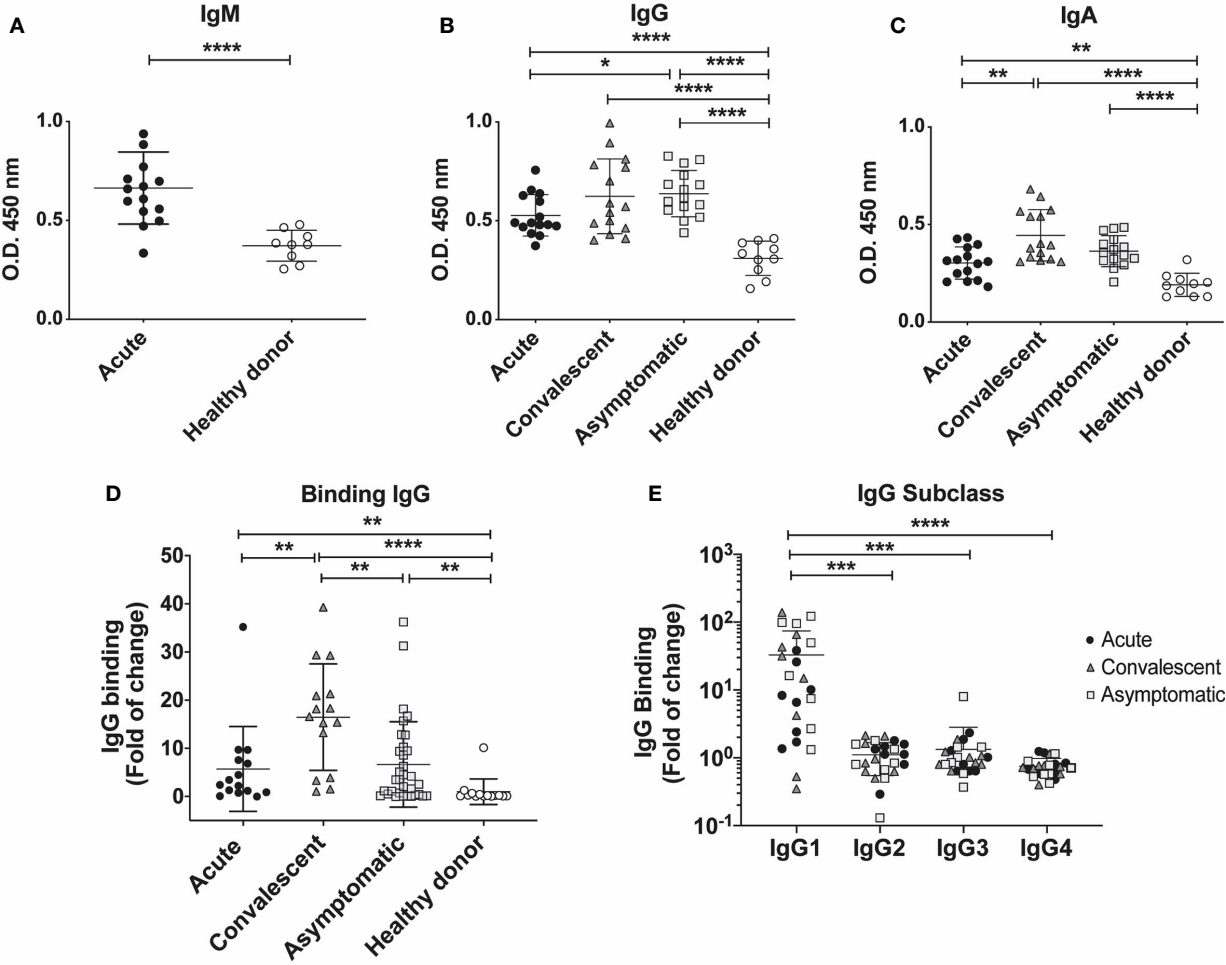
Serum antibody titers in individuals who have presented CHOV infection. **(A–C)** ELISA serological analysis displaying titers of IgM **(A)** IgG **(B)** and IgA **(C)** against CHOV-GP of acute patients (n=15), convalescent (n=15), asymptomatic (n=15) and healthy donors (n=10). **(D)** Cell-based binding assay to determine CHOV-GP specific IgG antibody binding by flow cytometry from serum of acute patients (n=15), convalescent (n=15), asymptomatic (n=33) and healthy donors (n=14). Each point on the graph represents a sample and the line the mean of the fold-increase obtained from each sample in duplicate. **(E)** Cell-based binding assay of CHOV-GP IgG subclasses by flow cytometry analysis of acute patients (n=8), convalescent (n=8), asymptomatic (n=8) and healthy donors (n=8). Results are shown in fold of increase in **(D, E)**. Error bars represent the SD of the mean. The mean comparison was performed with Mann-Whitney U test [p scales of < 0.0001, < 0.001 (***), < 0.01, < 0.05 (*)].

Serological analysis of antibody response against CHOV-GP was performed by ELISA. We observed moderate titers of IgM in the acute group as compared to healthy donors (**Figure 4A**). Next, we compared antibody titers against CHOV-GP between acute, convalescent and asymptomatic cohorts (**Figures 4B, C**). Results showed modest increased titers of IgG and low augmentation of IgA in the acute cohort compared to healthy donors. Moreover, convalescent serum samples showed higher titers of IgG and IgA against CHOV-GP compared to acute samples. Interestingly, asymptomatic seropositive individuals also presented significantly higher titers of IgG compared to healthy donors, showing high OD values similar to the ones found in the convalescent cohort (**Figure 4B**). In addition, IgA titers were also significantly higher in the asymptomatic group as compared to healthy donor samples (**Figure 4C**).

To assess the level of binding to cell surface expressed CHOV-GP, we examined the IgG binding using a cell based assay. CHOV-GPs 293T cells, which express the CHOV-GP at their surface, were incubated with purified polyclonal antibodies from acute, convalescent, asymptomatic and healthy donors. Our results showed a varied binding range of specific IgG antibodies against CHOV-GP, whereas total IgG isolated from healthy donors did not show reactivity. To select the samples with higher IgG titers to be used in further assays, we calculated the IgG binding as fold of change and defined as “positive” the samples with values higher than 5 binding-fold of specific IgG against CHOV-GP with respect to healthy donors (**Figure 4D**).

Comparing the CHOV-GP IgG antibody binding from individuals in different cohorts, we observed that convalescent individuals presented higher binding of anti-CHOV-GP IgG in serum (16.48 ± 11.04) when compared to the healthy donors (1.00 ± 2.65). A similar trend was detected in acute patients (5.72 ± 8.81) and asymptomatic seropositive individuals (6.65 ± 8.86) (**Figure 4D**).

In addition, we examined the subclass composition of the GP-specific IgG response in the serum of the different cohorts. Serum from convalescent subjects showed the presence of CHOV-GP specific IgG1 antibody. This CHOV-GP specific IgG1 was also detected in asymptomatic seropositive and acute individuals. IgG2, IgG3 and, IgG4 were not detectable, suggesting that IgG1 made up most of the response (**Figure 4E**). Whether or not this antibody response is correlated with protection should be tested.

### Antibody Neutralizing Titers of CHOV Positive Serum

It is well established that antibodies play a protective role against viral infections. To evaluate the protective efficacy of antibodies from the serum of different cohorts, a CHOV glycoprotein pseudotyped system was used to measured neutralization.

**Figure 5:**
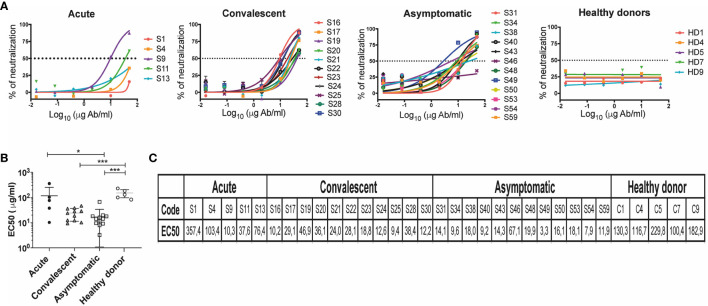
Neutralization titers in individuals who have presented CHOV infection. **(A)** Representative neutralization curves of acute patients (n=5), convalescent (n=11), asymptomatic (n=12) and healthy donors (n=5). The y-axis displays the percent pseudovirus neutralization, and the x-axis displays the reciprocal concentration Ab (log10). Each point on the graph represents the mean of the neutralization percentage obtained from each individual in triplicate. **(B)** The concentration of antibodies required to inhibit 50% of viral infection (EC50) was calculated based on antibody neutralization curves, EC50 (μg/ml) of different cohorts are shown. Error bars represent the SD of the mean. The mean comparison was performed with Mann-Whitney U test [p scales of < 0.001 (***), < 0.05 (*)]. **(C)** List of EC50 (μg/ml) of individuals included in the study.

In order to compare the neutralization efficacy of antibody response against CHOV across different cohorts, the concentration of antibodies required to inhibit 50% of viral infection (EC50) was calculated based on antibody titration curves (**Figure 5A**).

We measured the neutralizing capacity of samples that showed a 5-fold change in the IgG binding assay (**Figure 4D**). Presence of neutralizing antibodies in convalescent patients shown EC50 values between 9.36 and 46.94 μg/ml (n=11), and in the acute patients between 10.28 and 357.43 μg/ml (n=5). A large number of asymptomatic seropositive individuals also demonstrated the presence of neutralizing antibodies presenting EC50 values between 3.25 and 67.1 μg/ml (n=12). As expected, healthy donors did not show the presence of antibodies with anti-CHOV neutralizing activity (n=5) (**Figures 5A, B**).

Grouping of EC50 values by cohort revealed a higher neutralization potency in asymptomatic (seropositive) subjects (17.46 ± 16.37) when compared to healthy donors (152.0 ± 53.36), as well as in convalescent subjects (24.16 ± 12.73) and acute patients (117 ± 139.1) compared to healthy donors (**Figures 5B, C**).

### Clinical and Cytokine Association Analysis in Acute CHOV Infection

**Figure 6:**
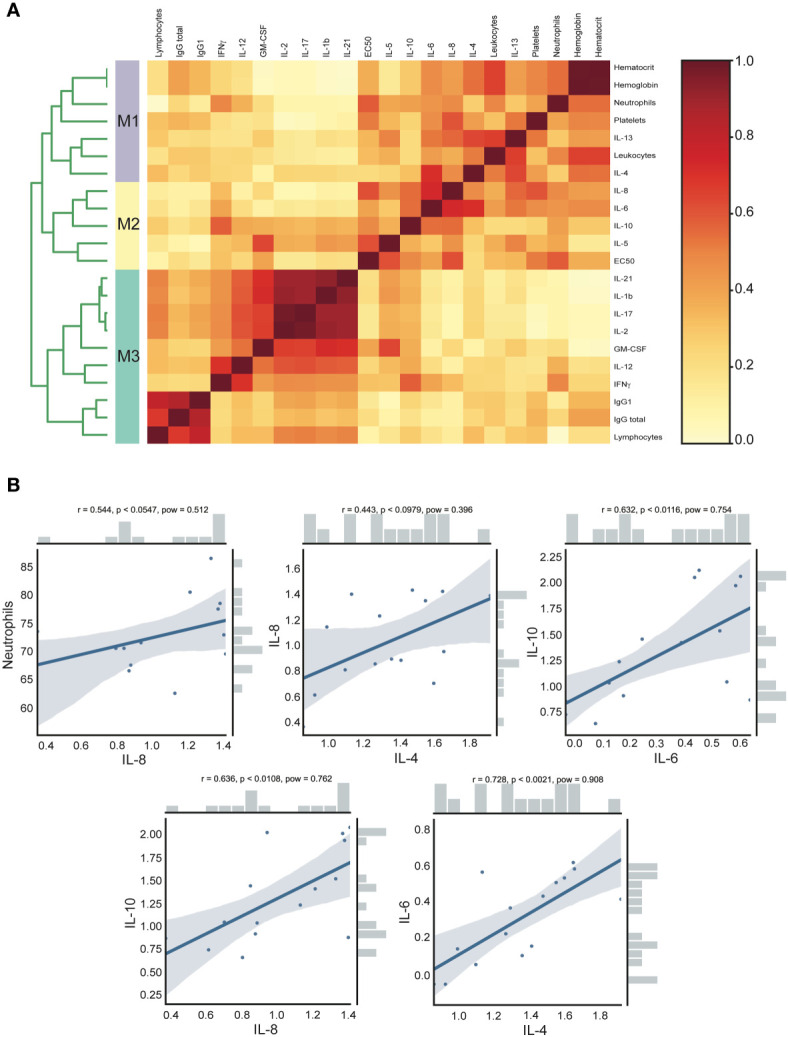
Clinical and cytokine concentration association analysis of acute CHOV infected individuals. **(A)** Heat map of cytokine and clinical parameters modules. The analysis was performed with the CytoMod method, the cytokine concentration was log-transformed and clustering was performed with the optimal number of modules (K)=3. Heat map color scale corresponds to the ratio of co-clustering. **(B)** Robust pairwise correlation. All pairwise parameters were calculated with a non-parametric percentage bend correlation and holm p- adjust value method. The correlation coefficient (r), the p-adjust (p), and the power (pow) values are shown at the top of each correlation plot. The bars shown in the side of the graph represent the distribution of the variables.

In order to detect associations and investigate the contribution of each cytokine with clinical parameters and also with humoral immune response in the acute group, we used an approach based on data clustering correlation to detect clinical parameters and cytokine associations using a previously described analysis named CytoMod (23). This approach increases the statistical power to detect associations (23), giving us a better understanding of the results and allowing integration of different variables. This analysis permitted the detection of a number of co-correlating cytokines and clinical parameters across acute CHOV infected individuals, which were grouped into distinct modules using hierarchical clustering. Cytokine concentration was analyzed in log values, resulting in an optimal number of calculated modules (K) of 3 (**Figure 6A**). The dendrogram analysis identified that within the acute group there were three clusters of associated variables: Module 1 (M1) includes the following clinical parameters: hematocrit, hemoglobin, neutrophils, platelets, leukocytes, and the cytokines IL-4, and IL13. Module 2 (M2) includes EC50 and the cytokines IL-8, IL-6, IL-10, and IL-5. Module 3 (M3) includes the clinical parameters: Lymphocytes, IgG total, IgG1, and the cytokines IL-21, IL-1β, IL-17, IL-2, GM-CSF, IL-12, and IFNγ.

Moreover, the pairwise correlation was calculated with a non-parametric percentage bend correlation and holm p-adjusted value method. The correlation coefficient (r), the p-adjust (p), and the power (pow) values are shown at the top of each correlation plot (**Figure 6B**). According to standard criteria (31) this analysis revealed a moderate positive correlation of neutrophils and IL-8 (r=0.544, p<0.0547), IL-8 and IL-4 (r=0.443, p<0.0979), IL-10 and IL-6 (r=0.632, p<0.0116), IL-10 and IL-8 (r=0.636, p<0.0108) and IL-6 with IL-4 (r=0.728, p<0.0021).

## Discussion

While most of the pathogenic NWH are characterized by inducing a severe illness with high morbidity and higher mortality rates, epidemiological studies indicate that CHOV infection causes a less harmful disease (6, 8, 10, 11). Little is known about the pathogenesis of CHOV infection; however, evidence found in studies related to other NWH infections indicates that immune response could be an important player in the pathogenesis of the disease (4).

In this study, we characterized the cytokines profile and antibody response elicited by CHOV infection from three different cohorts (acute, convalescent, asymptomatic/seropositive). Our findings indicate that high expression of Th1/Th2, as well as IL8 and IL-10 cytokines, were characteristic in most of the subjects tested during acute disease.

This reveals a coordinated immune response characterized by pro-inflammatory cytokines to fight and control infection accompanied by a strong anti-inflammatory signature that could lessen the symptomatology of this particular viral infection. Indeed, the data obtained in this study suggest that CHOV infected patients mount an early suppressive immune response potentially commanded by a Th2 immune profile that equilibrates the inflammatory signals observed as a measure of IFNγ, IL-6 among other inflammatory cytokines that rise later in acute infection. The finding is particularly interesting especially when we analyze the literature across different pathogenic NWHs infections that show stronger pro-inflammatory cytokine profiles characterized by IFNγ, TNFα, high levels of IL-6 as well as IL-1β (12–14, 16). Our finding that IL-4, IL-5, and IL-13 but also IL-10 are upregulated in the acute cohort compared to healthy subjects suggests that this anti-inflammatory phenotype could be responsible for the milder disease caused by CHOV infection. Indeed, Morzunov et al. (2015) show no differences in IL-4, IL-5, and IL-13 in a cohort of patients infected with pathogenic NWH from the United States (15).

Surprisingly, IL-4 was also found significantly upregulated across the asymptomatic cohort as well as in the convalescent individuals analyzed. These results point out the potential of IL-4 as a marker for CHOV infection; however, this hypothesis should be tested and validated in a larger cohort. IL-4 has not been proposed as a key cytokine in hantavirus infections. However, it has been evaluated in patients infected with OWHs where non-differences were observed between the cohort and the control samples (16). This is in stark contrast to our analysis. The same study showed that IL-4 was slightly upregulated in patients with HCPS from Argentina (16).

IL-4 is well characterized as a Th2 cytokine and also produced by CD4 T follicular helper cells with a major function in maintaining antibody development, class switching, and affinity maturation of antibodies (32). We did not find an association between IL-4 and serological IgG/IgG1, perhaps due to the low titers of the antibodies elicited against the GP during early infection. This finding raises a different hypothesis where IL-4 together with IL-10 could be the major driver to sustain strong suppression of the Th1 responses. This suppressive response could potentially give time to seroconvert and mount a proper antibody response to neutralize CHOV-GP. New evidence indicates that functional Th2-like T regulatory CD4 T cells (Treg) are present in peripheral tissues and could be a source of IL-4 (22, 33). In fact, a previous report has shown that respiratory syncytial virus (RSV) was able to induce a Th2-like phenotype in Treg cells in a mouse model of RSV infection (34).

Since IL-4 was found significantly upregulated during acute infection and also in the asymptomatic seropositive cohort, we analyzed potential association with other inflammatory cytokines. IL-4 was also found in association with IL-8 in acute infection. In line with this finding, other studies have shown that IL-8 rises early during infection, being responsible for neutrophil recruitment to the site of inflammation (35). A report studying HFRS also described that Puumala virus infection causes pro-inflammatory changes in endothelial cells, attracting and activating neutrophils to the endothelium surface by an IL-8 dependent mechanism (36). Furthermore, the fact that neutrophil depletion suppresses lung pathology in a mouse model for Hantaan virus certainly supports the idea of neutrophils as the driving force of hantavirus pathogenesis (37). Here we found a positive correlation between IL-8 and neutrophils in the acute cohort, however, this potential pathogenic phenotype could be being ameliorated by the strong Th2 immune phenotype found in patients with CHOV infection.

Another interesting finding corresponds to higher levels of IL-21 cytokine in the convalescent individuals analyzed, which is associated with high titers of neutralizing antibodies in convalescent HCPS individuals. Neutralizing antibodies were found years after disease presentation, which also has been reported for other NWH (20, 21).

During viral infection neutralizing antibody response is primarily directed against the envelope, thus we focus our study on the antibody response elicited against the glycoprotein. CHOV-specific IgM antibodies against glycoprotein appeared in high titers during the acute phase of infection. On the other hand, higher serum levels of IgG and IgA against CHOV-GP were detected in the convalescent and asymptomatic cohorts. Because the biological functions of IgG antibodies depend on their subclasses we also examined the different subtypes of IgG (38). The data indicate that most samples contained IgG1 subclass-specific to CHOV-GP throughout the illness, although this may be not the case for other hantaviruses (39). In fact, a previous report has described IgG3 as the major IgG subclass in antibody response to Sin Nombre virus infection (40). Interestingly, we found neutralizing antibody titers against CHOV-GP in a few acute samples, particularly in S9 and S11. In contrast, most asymptomatic samples presented high levels of neutralizing antibodies, with subject S49 presenting the highest titer of neutralizing antibodies among the cohorts. The finding that the asymptomatic cohort contains high titers of neutralizing antibodies together with upregulation of IL-4 supports the hypothesis that this group of previously infected people mounted a rapid and strong antibody response against CHOV-GP bypassing the pathogenic inflammatory process and consequently showing non signal of disease. Certainly, this hypothesis represents a difficult challenge to be tested.

The modular cytokine analysis allowed the identification of co-signaling cytokine associations, which may provide clues about the underlying immunological pathways occurring during acute CHOV infection. We found that modules 1 and 2 presented a stable association of cytokines and clinical parameters, unlike module 3. Visualized by the dendrogram (**Figure 6**), module 1 (M1) including IL-4, IL-13, hematocrit, hemoglobin, neutrophils, platelets and leukocytes was associated with module 2 (M2) that included IL-5, IL-6, IL-8, IL-10 and antibody EC50. The associations observed between these two modules highlight the fact that acute patients present a strong Th2 cytokine response associated with clinical parameters. In contrast, module 3 (M3) segregated in a different cluster of cytokines that include low IL-1β, IL-2, IL-12, moderate IL-17, IL-21, and high IFNγ, GM-CSF that were associated with low levels of lymphocytes and low titers of total IgG and IgG1 in the acute phase of infection.

Additionally, we found modestly increased levels of IL-6 in acute patients. IL-6 has been involved in the induction of acute inflammatory responses and has been described as a marker of severity (14, 15). In another report, IL-6 has been associated with IL-10 in viral infection (41), showing that IL-6 regulates the maturation of Treg cells and that IL-6 depletion also causes significant ablation of IL-10 in the lungs after RSV infection in an animal model (41). In line with that, our analysis indicates that IL-10 is significantly upregulated in acute patients, correlating positively with moderate titers of IL-6. Moreover, the early rise of IL-10 correlates positively with IL-8, among others proinflammatory cytokines and neutrophils levels. Interestingly, IL-6 correlates also with IL-4. Likewise, using profile correlation clustering we found an association between neutralizing antibody titers, Th2 cytokines and IL-10, reaffirming again the idea that these parameters could be involved in the mild disease presentation often observed in CHOV infection.

Certainly, this study contributes to extending our current knowledge regarding the immunological response in subjects with CHOV infection. From our knowledge, this is the first study that evaluates the immune serological profiles associated with hantavirus infection in Panama. We propose that the observed mixed profile of pro-, and anti-inflammatory cytokines observed in acute CHOV patients who resolve infection might be pivotal to control and suppress the strong inflammatory response occurring in the lungs. This could potentially explain the higher seroprevalence but lower severity observed in CHOV infection in Panama when compared with other NWHs at their endemic regions.

### Limitation of the Study

One of the limitations of the study was the low number of individuals analyzed, 15 acute, 15 convalescents, and 33 asymptomatic seropositive subjects. Another limitation was the availability of the samples, some of which were too limited to perform more assays. However, the results clearly show significant changes in the antibody and cytokine profile, giving us valuable information to establish a working hypothesis regarding the Th2 and regulatory signature that could potentially explain the mild type of disease frequently presented in individuals infected with CHOV.

## Data Availability Statement

The datasets presented in this article are not readily available because the datasets generated for this study are available on request to the corresponding authors. Requests to access the datasets should be directed to barmien@gorgas.gob.pa; mbarriac@udec.cl.

## Ethics Statement

Informed written consent was obtained from all adult participants and from parents or legal guardians of minors. Consent and assent forms were approved by Institutional Bioethics Committee at the Gorgas Memorial Institute of Health Studies in Panama City.

## Author Contributions

TPS designed and conducted experiments, analyzed data, and wrote the manuscript. JRS and PG analyzed data, sample collection and reviewed the manuscript. NZ and FF-V conducted experiments. FB analyzed data and review the manuscript. VF-L and AS-B performed clustering analysis and correlations. MC and RA provided feedback and reviewed the manuscript. JLG, BA, and MIB designed experiments and studied, wrote, reviewed and edited the manuscript. All authors contributed to the article and approved the submitted version.

## Funding

The work was financed by the Department of Research in Emergent and Zoonotic Diseases and the Ministry of Economy and Finance of Panama [grant number 111130150.501.274]. BA is a member of the SNI (Sistema Nacional de Investigación) from SENACYT (Secretaría Nacional de Ciencia y Tecnología) of Panama. The funders had no role in study design, data collection and analysis, decision to publish, or preparation of the manuscript. Also, this work was funded by SENACYT and IFARHU (Instituto para la Formación y Aprovechamiento de Recursos Humanos) of Panama to TS. This work was partially supported by the Chilean National Agency for Research and Development (ANID)/FONDEQUIP EQM150061.

## Conflict of Interest

JG, FB, and RA were partially supported by Ichor Biologics LLC.

The remaining authors declare that the research was conducted in the absence of any commercial or financial relationships that could be construed as a potential conflict of interest.
