# Supplementary material for: Cytokine Profiles and Antibody Response Associated to Choclo Orthohantavirus Infection
Source: Front Immunol. 2021 Mar 19;12:603228. doi: 10.3389/fimmu.2021.603228 (PMC8017165; doi:10.3389/fimmu.2021.603228)
Supplement: Supplementary file 1 [file DataSheet_1.pdf]

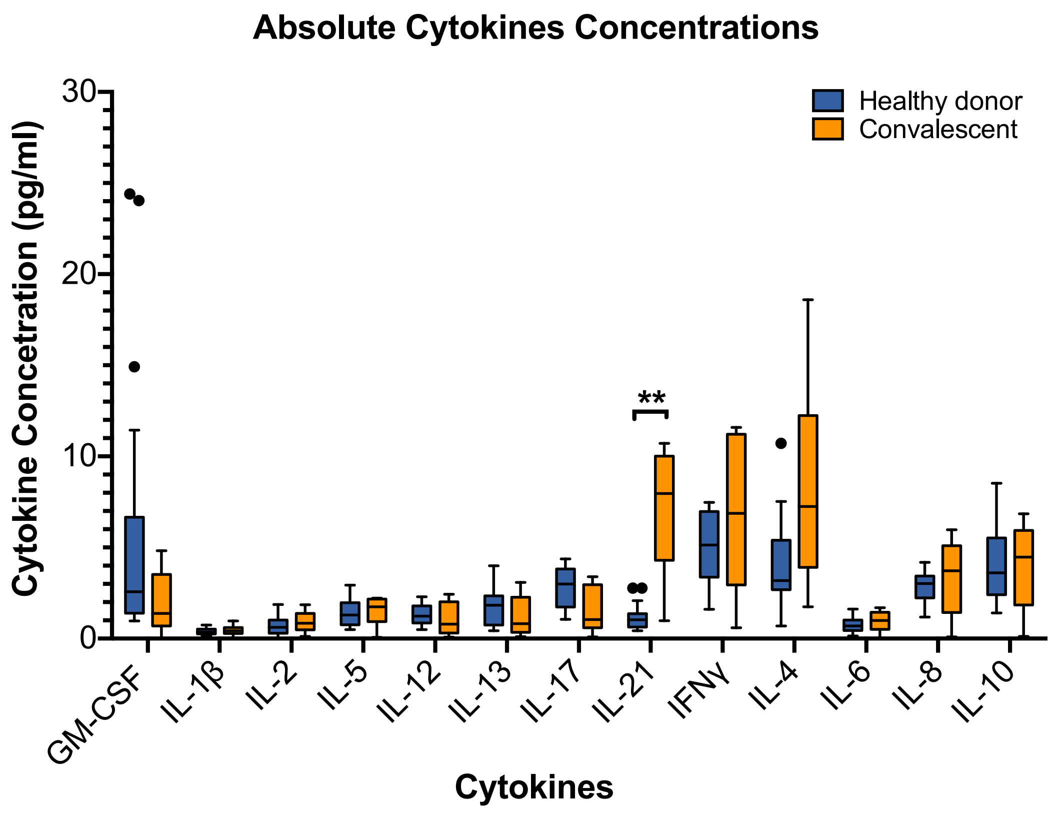

Supplementary Figure 1A. Box plot of serum cytokine concentrations in pg/ml of convalescent (n=7) and healthy donors (n=21) used as controls. The box plot diagram is displayed by quartiles and the middle line represents the mean concentration. Error bars represent the SD of the mean. Black dots correspond to out layers. P value **0,0014 by Mann-Whitney test.

Figure 1B. Cytokine concentration in serum of healthy donor and convalescent subjects.

	Healthy Donor (n=21)	Convalescent (n=7)
Cytokine	mean ± SD (pg/ml)	mean ± SD (pg/ml)
GM-CSF	5,70 ± 7,14	1,94 ± 1,74
IL-1β	0,39 ± 0,17	0,45 ± 0,31
IL-2	0,70 ± 0,46	0,92 ± 0,59
IL-5	1,36 ± 0,72	1,53 ± 0,82
IL-12	1,36 ± 0,56	1,06 ± 0,93
IL-13	1,77 ± 1,04	1,21 ± 1,13
IL-17	2,75 ± 1,08	1,52 ± 1,29
IL-21	1,16 ± 0,68	7,13 ± 3,57
IFNγ	5,01 ± 1,83	6,79 ± 4,50
IL-4	4,11 ± 2,43	8,27 ± 5,80
IL-6	0,73 ± 0,37	0,96 ± 0,58
IL-8	2,87 ± 0,81	3,37 ± 2,14
IL-10	3,90 ± 1,98	4,00 ± 2,51
